# Supplementary material for: Nonequilibrium dynamics of spontaneous symmetry breaking into a hidden state of charge-density wave
Source: Nat Commun. 2021 Jan 25;12:566. doi: 10.1038/s41467-020-20834-5 (PMC7835373; doi:10.1038/s41467-020-20834-5)
Supplement: Supplementary file 1 — Supplementary Information [file 41467_2020_20834_MOESM1_ESM.pdf]

Supplementary Information for

Nonequilibrium dynamics of spontaneous symmetry breaking into a hidden state of charge-density wave

Faran Zhou[1], Joseph Williams[1], Shuaishuai Sun[1], Christos D. Malliakas[2,3], Mercouri G. Kanatzidis[2,3], Alexander F. Kemper[4], Chong-Yu Ruan[1*]

[1] Department of Physics and Astronomy, Michigan State University, East Lansing, MI 48824, USA.

[2] Department of Chemistry, Northwestern University, Evanston, IL 60208, USA.

[3] Materials Science Division, Argonne National Laboratory, Argonne, IL 60439, USA.

[4] Department of Physics, North Carolina State University, Raleigh, NC 27695, USA

* e-mail: ruanc@msu.edu

This Supplementary Information includes:

Supplementary Figure 1: The laser field profile within the $CeTe_3$ film

Supplementary Figure 2: Thermal phase transition of CDW state in $CeTe_3$

Supplementary Figure 3: The fluence-dependent c-CDW scattering amplitude evolution

Supplementary Figure 4: Peak profile fitting for extracting correlation length ξ

Supplementary Figure 5: Theoretical modeling of CDW diffraction pattern

Supplementary Figure 6: Theoretical calculations of susceptibility (χ) and nesting vector (q_χ)

Supplementary Note 1: Non-equilibrium scattering formalism

Supplementary Note 2: Refinement and modeling

Supplementary Note 3: Connection to ARPES experiments: theoretical calculations

References

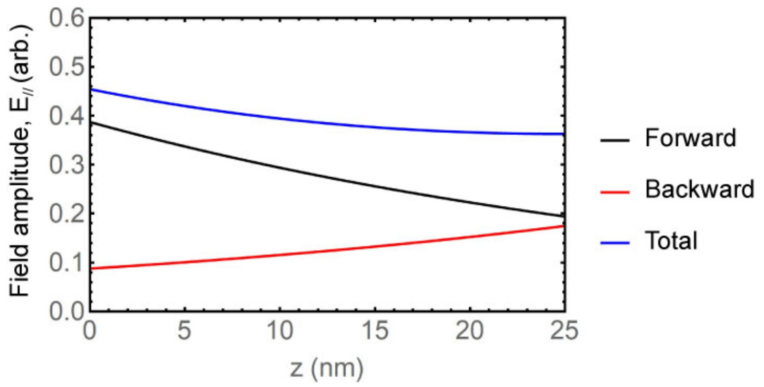

Supplementary Figure 1. The laser field profile within the $CeTe_3$ film | The strength of the exciting laser field present in the film. The black and red represents the field magnitude associated with the forward and backward moving components (the phases of the two components are not the same here), which add together to give the total field present at each location.

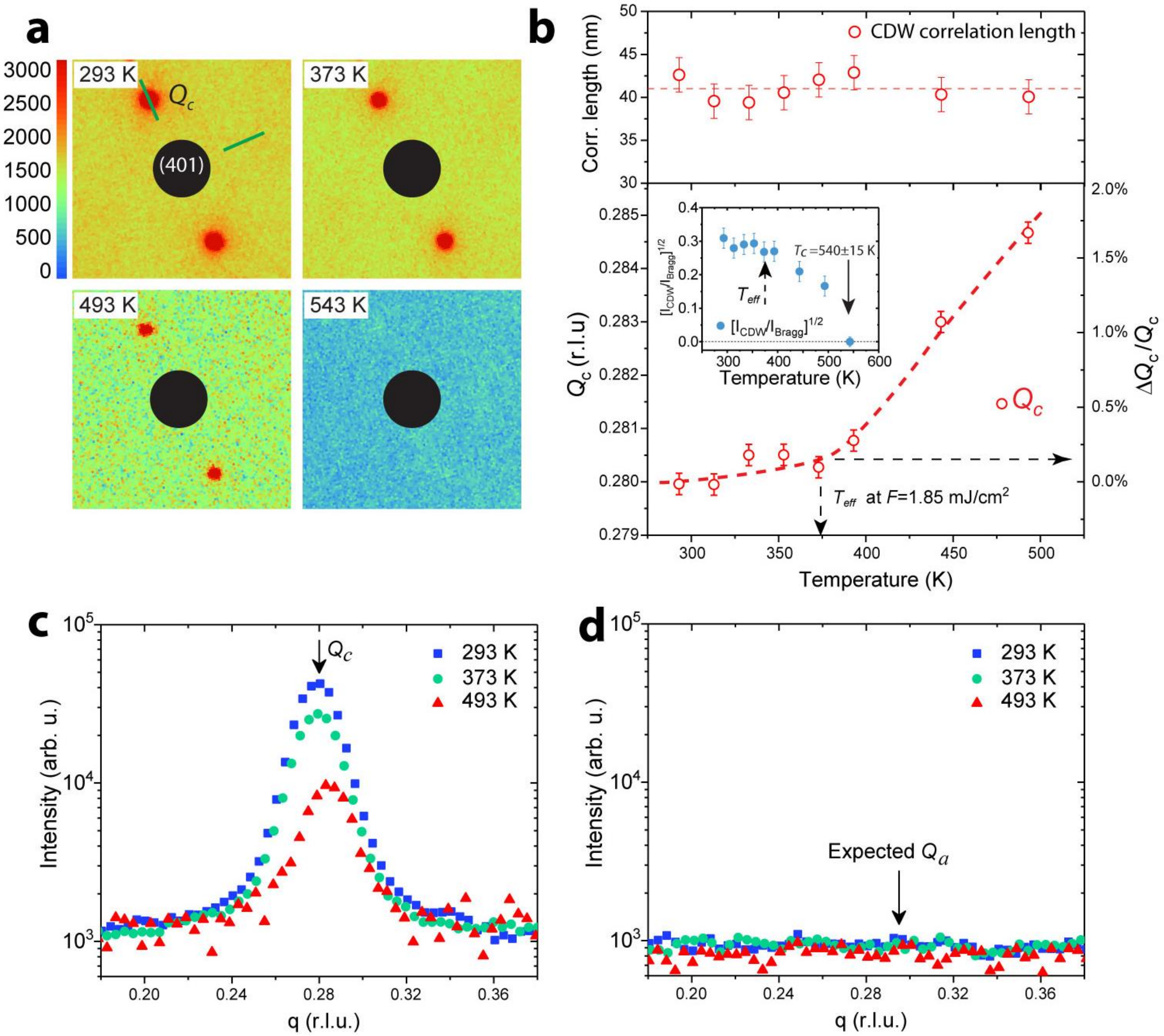

Supplementary Figure 2. Thermal phase transition of CDW state in $CeTe_3$ | a. The diffraction images showing the CDW satellite peaks evolution at selected temperatures near phase transition obtained from a transmission electron microscope (JEOL 2010F) using the Gatan OneView 4k×4k camera. **b.** Top panel shows the CDW correlation length evolution as the temperature is increased from room temperature. The bottom panel shows the corresponding shift of the CDW wavevector ($\mathbf{Q}_c$). The inset shows the corresponding relative changes in the CDW satellite intensity, approaching the melting temperature T_c=540±15 K. The error bars are the standard deviations from the fittings. **c & d.** The line profiles along the $\mathbf{Q}_c$ and $\mathbf{Q}_a$ (expected) directions at three selected temperatures.

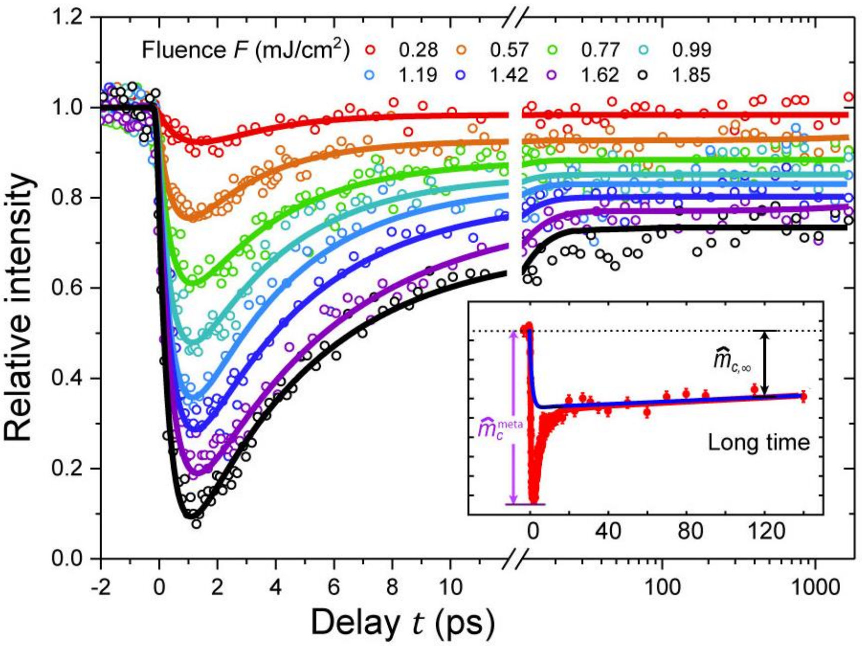

Supplementary Figure 3. The fluence-dependent *c*-CDW scattering amplitude evolution | The inset shows the two different amplitudes representing the nonthermal ($\hat{m}_c^{meta}$) and thermal ($\hat{m}_{c,\infty}$) responses to the laser excitation.

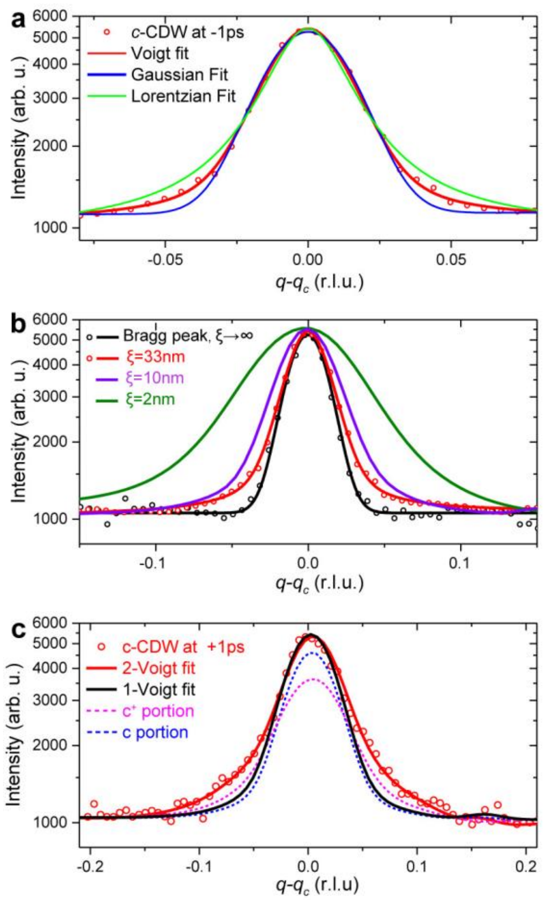

Supplementary Figure 4. Peak profile fitting for extracting correlation length ξ | a, The elastic profile (circle) taken from a *c*-CDW satellite peak at -1ps is best fitted with a Voigt function (red), in comparison with the Gaussian (blue), and Lorentzian (green) functions. **b,** Retrieval of the Lorentz parameters (amplitude and correlation length) through fitting with the Voigt function. The instrumental response is determined from a near-by lattice Bragg peak fitted with a Gaussian (in black). For better comparison, the central amplitudes of the profiles are scaled to the same value. **c,** Intensity profile of *c*-CDW at +1 ps, where a single-Voigt function (black curve) cannot fit the peak well. The two-Voigt function fitting is required to reach a good agreement. Here, the blue dashed line represents the suppressed initial *c*-CDW order and the pink dashed line represents the c^+ portion. Red solid line is sum of the two dashed portions.

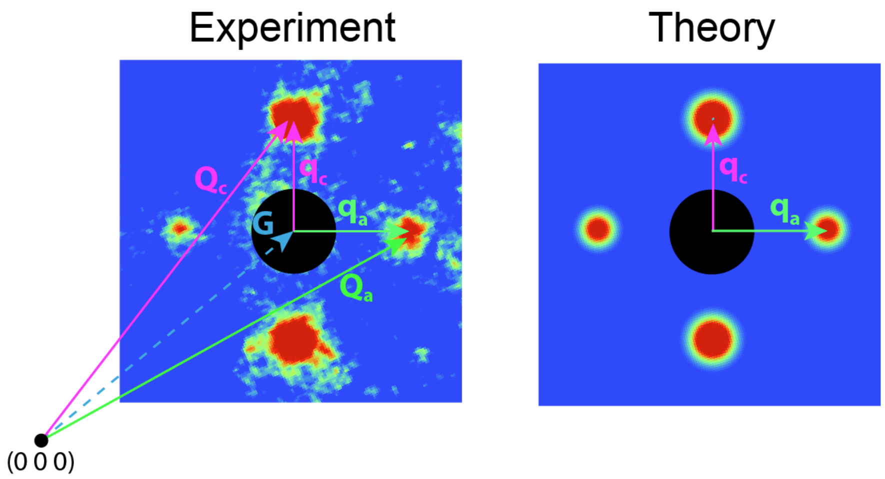

Supplementary Figure 5. Theoretical modeling of CDW diffraction pattern | The scattering pattern is reconstructed based on the Fourier Transform of the real-space correlation function.

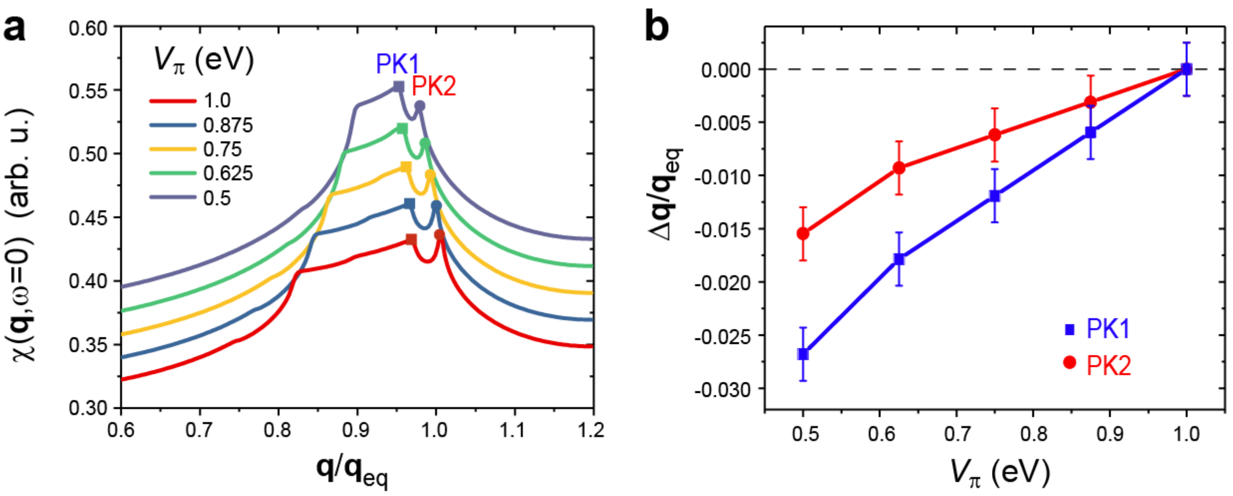

Supplementary Figure 6. Theoretical calculations of susceptibility (χ) and nesting vector ($\mathbf{q}_\chi$) | a, $\chi(\mathbf{q}, \omega = 0)$ along $\mathbf{q_x} = \mathbf{q_y}$ (offset for clarity). Markers indicate the two local maxima in the susceptibility. The horizontal axis is scaled to the absolute maximum for V_π = 1.0 eV. **b,** Peak position of the charge susceptibility along the (11) direction. The error bars arise from the finite momentum resolution of the calculation.

Supplementary Note 1. Non-equilibrium scattering formalism

To derive the time-dependent structure factor for a lattice with lattice distortion wave (LDW) considering the fluctuation effects, we write $S(\mathbf{q}, t) = \int e^{-i\boldsymbol{q}\cdot(\mathbf{r}'-\mathbf{r}'')}\langle\rho(\boldsymbol{r}', t)\rho(\boldsymbol{r}'', t)\rangle\, d\mathbf{r}' d\mathbf{r}''$, where $\rho(\boldsymbol{r}, t)$ is the density function and $\langle\rho(\mathbf{r}', t)\rho(\mathbf{r}'', t)\rangle$ is the equal-time density correlation function of the atomic lattice with the bracket denoting the spatial and ensemble averaging of the non-equilibrium system. In this expression, the $\rho(\boldsymbol{r}, t)$ of a dynamical state is modulated by the displacement vector $\mathbf{u_L}(\mathbf{r}, t) = \mathbf{u}_q(\mathbf{r}, t) + \mathbf{u}_\eta(\boldsymbol{r}, t)$ at each lattice site $\mathbf{L}$. The first term, $\mathbf{u_q}(\boldsymbol{r}, t) = \sum_{\boldsymbol{q}} u_{0,\boldsymbol{q}}\hat{\mathbf{e}}_{\boldsymbol{q}} sin\left(\mathbf{q}\cdot\mathbf{r} - \omega_{\boldsymbol{q}} t + \phi_{\boldsymbol{q}}\right)$, includes contributions from phonons at different momenta $\mathbf{q}$ (soft modes included); meanwhile, the contribution associated with the singe-wavevector LDW is expressed in $\mathbf{u}_\eta(\mathbf{r}, t) = u_{0,\eta}\hat{\mathbf{e}}_\eta \left(1 + \delta u_\eta(\mathbf{r}, t)\right) sin\left[\mathbf{Q_\eta}\cdot\mathbf{r} + \delta\phi_\eta(\mathbf{r}, t)\right]$, where the amplitude and phase fluctuations, $\delta u_\eta(\mathbf{r}, t) = \sum_{\boldsymbol{k}} u_{0,\boldsymbol{k}} sin(\mathbf{k}\cdot\mathbf{r} - \omega_{\boldsymbol{k}} t)$ and $\delta\phi(\mathbf{r}, t) = \sum_{\boldsymbol{k}} \phi_{0,\boldsymbol{k}} sin(\mathbf{k}\cdot\mathbf{r} - \omega_{\boldsymbol{k}} t)$, are expanded in series of fluctuation waves at momentum $\mathbf{k}$ and contribute to diffusive scattering $S_{Q,k}$. With the density function $\rho(\boldsymbol{r}, t)$ modulated by this general expression of $\mathbf{u_L}(\mathbf{r}, t)$, we can derive the respective structure factors S_G, S_Q and $S_{Q,k}$ to see how they are impacted by the fluctuation effects. We obtain

$$\begin{cases} S_G(\mathbf{q}) = \delta(\mathbf{q} - \mathbf{G}_{hkl})|f_L|^2 e^{-2M_{hkl}} \left| J_0\left(\mathbf{G}_{hkl} \cdot \hat{\mathbf{e}}_\eta u_{0,\eta}(t)\right)\right|^2 , \\ S_Q(\mathbf{q}) = \delta(\mathbf{q} - \mathbf{G}_{hkl} - \mathbf{Q})|f_L|^2 e^{-2M_{hkl}} \left| J_1\left(\mathbf{G}_{hkl} \cdot \hat{\mathbf{e}}_\eta u_{0,\eta}(t)\right)\right|^2 \prod_{\boldsymbol{k}} |J_0(\phi_{\boldsymbol{k}})|^2 , \\ S_{Q,k}(\mathbf{q}) = \delta(\mathbf{q} - \mathbf{G}_{hkl} - \mathbf{Q} - \mathbf{k})|f_L|^2 e^{-2M_{hkl}} \left| J_1\left(\mathbf{G}_{hkl} \cdot \hat{\mathbf{e}}_\eta u_{0,\eta}(t)\right)\right|^2 |J_1(\phi_{\boldsymbol{k}})|^2 , \end{cases} \quad (1)$$

where f_L represents the scattering amplitude from the lattice unit cell and J is the Bessel function. The excitation of phonons leads to the damping term $e^{-2M_{hkl}(t)}$, the commonly known Debye-Waller factor (DWF) in the equilibrium condition. In describing the non-equilibrium process, the role of DWF can be viewed form the expressive form $2M_{hkl} = \sum_{\boldsymbol{q}} \frac{1}{2}\left(\mathbf{G}_{hkl} \cdot \hat{\mathbf{e}}_{\mathbf{q}} u_{0,\mathbf{q}}(t)\right)^{\mathbf{2}}$, which is the sum of the mean-squared (*ms*) displacements along $\mathbf{G}_{hkl}$ from all active phonons in the system. Since laser pumping primarily couple to the gapped states tied to CDW formation, so by examining the difference introduced in $2M_{hkl}$ after the pumping, one can isolate the key contributions from the soft modes critically involved in the non-thermal phase transition. On the phase ordering dynamics, one can easily see from the 2nd and 3rd equations that exciting collective modes at momentum $\mathbf{k}$ transfers the scattering weight from S_Q, with a decay of $|J_0(\phi_{\boldsymbol{k}})|^2$, into $S_{Q,k}$ with an increase of $|J_1(\phi_{\boldsymbol{k}})|^2$. In this regard, we consider mainly the phase modes[1]; nonetheless, similar results can be derived for the amplitude modes as well. It can be shown that by fully accounting the diffuse scattering contributions (including higher order fluctuations) the integrated intensity $m_{\boldsymbol{Q}}(t) = \int \left(S_{\boldsymbol{Q}}(\mathbf{k}) + S_{\mathbf{Q},\mathbf{k}}(\mathbf{k})\right) d\mathbf{k} = |f_{\mathbf{L}}|^2 e^{-2M} \left| J_1\left(\mathbf{G}_{hkl} \cdot \hat{\mathbf{e}}_\eta u_{0,\eta}(t)\right)\right|^2$ is unchanged in spite of exciting collective modes. This sum rule allows one to deduce the order parameter magnitude $u_{0,\eta}(t)$ directly from the $m_{\boldsymbol{Q}}(t)$. As shown below, what makes the analyses apparent is that the modification of S_Q introduced by the lattice DWF is generally much smaller than the change from the order parameter evolution during the phase transition. This makes it easy to separate the order parameter evolution from the lattice phonon dynamics. Rigorously, one can independently retrieve the respective order parameter dynamics via calculating $h(t) = \frac{m_{Q_l}(t)}{m_G(t)} = \frac{\left|J_1\left(\mathbf{G}_{\mathrm{hkl}} \cdot \hat{\mathbf{e}}_{\eta_l} u_{0,\eta_l}(t)\right)\right|^2}{\prod_l \left|J_0\left(\mathbf{G}_{hkl} \cdot \hat{\mathbf{e}}_{\eta_l} u_{0,\eta_l}(t)\right)\right|^2}$ and $g(t) = \frac{m_{Q_a}(t)}{m_{Q_c}(t)} = \left|\frac{J_1(\mathbf{G}_{hkl} \cdot \hat{\mathbf{e}}_{\eta_a} u_{0,\eta_a})}{J_1(\mathbf{G}_{hkl} \cdot \hat{\mathbf{e}}_{\eta_c} u_{0,\eta_c})}\right|^2$. Given the orthogonal nature of two order parameters, one can choose the Bragg peak to be along [001] or [100] for isolating the contribution from one specific CDW system to determine the respective $u_{0,\eta_l}(t)$. Then the soft mode dynamics can be determined from analyzing the normalized DWF: $l_{hkl}(t) = \frac{e^{-2M_{hkl}(t)}}{e^{-2M_{hkl}(t<0)}} = e^{-2\Delta M_{hkl}(t)}$ taken at different $\mathbf{G}_{hkl}$ as shown in the text.

Supplementary Note 2. Refinement and modeling

A data analysis program is developed for this study, aiming to refine the key CDW parameters based on a structure model. We start from constructing the correlation function $S_{\eta}(\mathbf{r},t) \approx \langle u_{\eta}(\mathbf{r},t) u_{\eta}(\mathbf{0},0)\rangle$, where $u_{\eta}(\mathbf{r},t)$ represents the LDW and is related to the CDW order parameter by the relationship $\eta = u_{\eta}/A_{u\eta}$. After ensemble-averaging and with l denotes a, c, or c^{+}-CDW, an effective expression of the LDW to include the finite correlation length (ξ_l) is given

$$u_l(\mathbf{r},t) = u_{0,\eta_l} e^{i(\boldsymbol{Q}_l(t)\cdot\mathbf{r}+\phi_l)} e^{-\frac{|r-\boldsymbol{r_0}|}{2\xi_l(t)}}, \qquad (2)$$

centered around a reference position $\mathbf{r_0}$. The satellite structure factor $S_{Q_l}(\mathbf{q},t)$ is just the Fourier counter part of the correlation function, i.e.:

$$S_{Q_l}(\mathbf{q},t) = \int d\mathbf{r}\, e^{\boldsymbol{i}\mathbf{q}\cdot\mathbf{r}} S_{\eta_l}(\mathbf{r},t), \qquad (3)$$

which can be calculated based on $u_l(\boldsymbol{r},t)$. The first inputs to different LDW states at different momenta, including parameters: u_{0,η_l}, $\mathbf{Q}_l$, and ξ_l, are determined by fitting $S_{Q_l}(\mathbf{q},t)$, taking the form of a Voigt function – a convolution of a Lorentzian and the instrument Gaussian response, $G(\mathbf{q})$. The correlation length is calculated from $\xi_l = 1/w_l$ with w_l the Lorentzian width at half-width-at-half-maximum. The data refinement is based on simulating the satellite diffraction patterns:

$$S_{theo}(\mathbf{q},t) \approx FFT\{S_{tot}(\mathbf{r},t)\} \otimes G(\mathbf{q}), \qquad (4)$$

where FFT represents the Fast Fourier Transform and $G(\mathbf{q})$ is the Gaussian convolution function represents the instrument limit of the detector. $S_{tot}(\mathbf{r},t)$ is the autocorrelation function of the displacement field, which is simply $|\mathbf{u}_{tot}(\mathbf{r},t)|^2$ with $\mathbf{u}_{tot}(\mathbf{r},t) = \sum_l \mathbf{u}_{\eta_l}(\mathbf{r},t)$ to include the contribution from different states. As discussed in the text, bi-directional order emerges in the existing c-CDW domain. This is taken account by centering the $\mathbf{u}_{\eta_l}(\mathbf{r},t)$ of all three CDW branches at the same location. The phase of the a and c^{+} branches are kept the same. While in theory, the phase of c-CDW is uncorrelated with the emerging order, for simplicity it is chosen to be the same as it does not significantly affect the result.

The refinement is conducted iteratively. The fitting to obtain the initial input of u_{0,η_l}, $\mathbf{Q}_l$, and ξ_l from the $S_{Q_l}(\mathbf{q},t)$ with a Voigt function is shown in Supplementary Fig. 4. Here, $G(\mathbf{q})$ is determined in situ through fitting the neighboring lattice Bragg peak given that correlation length of the lattice exceeds the instrument limit (≈40 nm), see Supplementary Fig. 4b. The width of $G(\mathbf{q})$, σ_G typically ≈ 0.018Å^{-1}, is held constant over the fitting. To consider the second (c^{+}) component in the c-axis ordering, we include a second Voigt function in fitting the $S_{Q_c}(\mathbf{q},t)$ profile. The need to include the additional component is featured in Supplementary Fig. 4c. In fitting the intensity profile at 1 ps (red open circles), the data cannot be reconciled with just one Voigt function. In the fitting procedure, the background is removed via minimizing the non-negativeness with a linear polynomial.

The refinement shown in Fig. 4b is based on comparing the theoretical line profiles, extracted from the 2D diffraction recreated by Supplementary Eqn. (4), with the data profiles. In this procedure, higher order diffraction, such as the satellite peak at $c^* - 2Q_c$ in Fig. 4b, can be reproduced. The model is better constrained by fitting the vertical and transverse profiles at the same time. This better accounts for the diffuse scattering as the background level is similar in both directions. In the data presented in Fig. 4b near the phase transition, the error bars for the peak position is 0.002 r.l.u., and the intensity about 5%. The Supplementary Fig. 5 shows the comparison between the theoretical diffraction pattern deduced from the refinement procedure and the experiments.

Supplementary Note 3. Connection to ARPES experiments: theoretical calculations

Here, we will show that the reduction of the inter-orbital coupling $t_\perp$ leads to a reduction of the nesting vector $\mathbf{q}_{\boldsymbol{\chi}}$. We evaluate the pairing vector by calculating the static charge fluctuation susceptibility $\chi(\boldsymbol{q}, \omega = 0)$ based on a model band structure that incorporates the reduction in $t_\perp$. Although $\chi(\mathbf{q}, \omega = 0)$ has peaks at several potential nesting vectors, a focused electron-phonon coupling vertex selects a particular one ($\mathbf{q}_{\boldsymbol{\chi},\mathbf{1}}$) of these (see Fig. 4 in Eiter et al.[2]). Here, we focus on the underlying susceptibility and its behavior as the V_π ($\propto t_\perp$) is reduced. Supplementary Fig. 6a shows line cuts of the focused $\chi(\mathbf{q}, \omega = 0)$ along the (11) direction (where the electron-phonon coupling vertex is peaked) for several values of V_π. For both the local maxima, as V_π is reduced, the peak in the susceptibility shifts towards lower $\mathbf{q}$ values, which could explain the increase in CDW ordering wave-vector $\mathbf{Q_c}$ observed here – since $\mathbf{Q_c} = \mathbf{c}^* - \mathbf{q}_{\boldsymbol{\chi},\mathbf{c}}$. Supplementary Fig. 6b shows the obtained peak value as a function of V_π.

To evaluate the theoretical ordering vector, we follow the methods outlined in Eiter et al.[2] The charge susceptibility is defined as

$$\chi(\mathbf{q}, \omega) = 2 \sum_{\alpha,\beta\in\pm} \sum_{\boldsymbol{k}} \frac{f\left(\epsilon^{\alpha}_{\boldsymbol{k+q}}\right) - f\left(\epsilon^{\beta}_{\boldsymbol{k}}\right)}{\omega + \epsilon^{\alpha}_{\boldsymbol{k+q}} - \epsilon^{\beta}_{\boldsymbol{k}} + i0^+}, \tag{5}$$

where $f(x)$ is the Fermi function, $\epsilon^{\alpha}_{\boldsymbol{k}}$ is the bare dispersion for the αth band at momentum $\mathbf{k}$. Both intra- and inter-band susceptibilities are included. The tight-binding model is based on the one used in Eiter et al.[2]. Specifically, at a 25% reduction in V_π reported by ARPES at F=0.9 mJ/cm^2, the CDW ordering vector $\mathbf{Q_c}$ should increase by ≈1.25%. This is consistent with the experiments here; at F=1.85 mJ/cm^2, a maximum $\mathbf{Q_c}$ shift of ≈2.5 % is observed; see Fig. 4d.

References

1. Overhauser, A. W. Observability of Charge-Density Waves by Neutron Diffraction. *Physical Review B* **3**, 3173-3182, doi:10.1103/PhysRevB.3.3173 (1971)
2. Eiter, H.-M. *et al.* Alternative route to charge density wave formation in multiband systems. *Proceedings of the National Academy of Sciences of the United States of America* **110**, 64-69 (2013)
